# Machine Learning Analysis of Predictors for Inhaled Nitric Oxide Therapy Administration Time Post Congenital Heart Disease Surgery: A Single-Center Observational Study

**DOI:** 10.7759/cureus.65783

**Published:** 2024-07-30

**Authors:** Shuhei Niiyama, Takahiro Nakashima, Kentaro Ueno, Daisuke Hirahara, Masatoyo Nakajo, Yutaro Madokoro, Mitsuhito Sato, Kenshin Shimono, Takahiro Futatsuki, Yasuyuki Kakihana

**Affiliations:** 1 Department of Emergency and Intensive Care Medicine, Kagoshima University Hospital, Kagoshima, JPN; 2 Department of Clinical Engineering, Kagoshima University Hospital, Kagoshima, JPN; 3 Department of Pediatrics, Graduate School of Medical and Dental Sciences, Kagoshima University, Kagoshima, JPN; 4 Department of Management Planning Division, Harada Academy, Kagoshima, JPN; 5 Department of Radiology, Graduate School of Medical and Dental Sciences, Kagoshima University, Kagoshima, JPN

**Keywords:** machine learning, open heart surgery, congenital heart disease, inhaled nitric oxide, pulmonary hypertension

## Abstract

Background

Congenital heart disease (CHD) is a structural deformity of the heart present at birth. Pulmonary hypertension (PH) may arise from increased blood flow to the lungs, persistent pulmonary arterial pressure elevation, or the use of cardiopulmonary bypass (CPB) during surgical repair. Inhaled nitric oxide (iNO) selectively reduces high blood pressure in the pulmonary vessels without lowering systemic blood pressure, making it useful for treating children with postoperative PH due to heart disease. However, reducing or stopping iNO can exacerbate postoperative PH and hypoxemia, necessitating long-term administration and careful tapering. This study aimed to evaluate, using machine learning (ML), factors that predict the need for long-term iNO administration after open heart surgery in CHD patients in the postoperative ICU, primarily for PH management.

Methods

We used an ML approach to establish an algorithm to predict 'patients with long-term use of iNO' and validate its accuracy in 34 pediatric postoperative open heart surgery patients who survived and were discharged from the ICU at Kagoshima University Hospital between April 2016 and March 2019. All patients were started on iNO therapy upon ICU admission. Overall, 16 features reflecting patient and surgical characteristics were utilized to predict the patients who needed iNO for over 168 hours using ML analysis with AutoGluon. The dataset was randomly classified into training and test cohorts, comprising 80% and 20% of the data, respectively. In the training cohort, the ML model was constructed using the important features selected by the decrease in Gini impurity and a synthetic oversampling technique. In the testing cohort, the prediction performance of the ML model was evaluated by calculating the area under the receiver operating characteristics curve (AUC) and accuracy.

Results

Among 28 patients in the training cohort, five needed iNO for over 168 hours; among six patients in the testing cohort, one needed iNO for over 168 hours.

CPB, aortic clamp time, in-out balance, and lactate were the four most important features for predicting the need for iNO for over 168 hours.

In the training cohorts, the ML model achieved perfect classification with an AUC of 1.00. In the testing cohort, the ML model also achieved perfect classification with an AUC of 1.00 and an accuracy of 1.00.

Conclusion

The ML approach identified that four factors (CPB, in-out balance, aortic cross-clamp time, and lactate) are strongly associated with the need for long-term iNO administration after open heart surgery in CHD patients. By understanding the outcomes of this study, we can more effectively manage iNO administration in postoperative open heart surgery in CHD patients with PH, potentially preventing the recurrence of postoperative PH and hypoxemia, thereby contributing to safer patient management.

## Introduction

Pulmonary hypertension (PH) in congenital heart disease (CHD) is typically due to left-to-right shunt defects or postcapillary hypertension resulting from left heart obstructive disease [1]. Common defects include ventricular septal defects, atrial septal defects, and remnants of the ductus arteriosus [1]. Although some individuals are born with advanced pulmonary vascular disease, available data suggest that early treatment, including surgical intervention, may prevent the subsequent development and progression of PH and reduce the burden of pulmonary vascular disease [2]. In the Fontan circulation, elevated pulmonary vascular resistance (PVR) is the primary cause of Fontan insufficiency [3]. Pulmonary arterial pressure in the Fontan circulation almost never reaches the threshold used to define PH in patients with biventricular circulation, where the mean pulmonary arterial pressure is >20 mmHg. In Fontan patients, a transpulmonary gradient >6 mmHg and a PVR index >3 WU × m2 indicate pulmonary hypertensive vascular disease, as suggested by the European Pediatric Pulmonary Vascular Disease Network [4]. Although its pathophysiology is unknown, evidence suggests it may be caused by a lack of pulsatile flow [5] or thromboembolism [6] due to endothelial dysfunction in the pulmonary vasculature. Furthermore, PH therapy for patients after Fontan repair is intended to improve quality of life, control heart failure, and improve exercise tolerance [7].

The hallmark of this disorder in the early postoperative period is a postoperative pulmonary hypertensive crisis, characterized by an acute increase in PVR that initiates a cycle of right ventricular failure and poor cardiac output. If left untreated, cardiac arrest and death may follow [8]. Furthermore, PHTC is a major cause of morbidity and mortality following congenital heart surgery. Inhaled nitric oxide (iNO) is used as rescue therapy, which is a widely accepted standard of care for PH and has been studied in the context of cardiac surgery for CHD [9].

Recent advances in ML have led to an increase in the development of predictive algorithms using ML approaches in the intensive care field [10]. In Japan, iNO is administered during the perioperative period of cardiac surgery. However, reducing or discontinuing iNO can worsen postoperative PH and hypoxemia. Therefore, carefully administering and gradually tapering iNO while maintaining strict circulatory management is crucial. In Japan, iNO is indicated during the perioperative period of cardiac surgery, but it is not well understood which patients require careful iNO management, and there is little evidence to guide optimal weaning procedures. Once oxygenation improves, iNO can be weaned relatively rapidly to 5 ppm without difficulty and discontinued within five days. Therefore, we used ML to establish an algorithm and validate its accuracy for predicting the long-term use of iNO (>168 h) in patients admitted to the ICU after pediatric open heart surgery.

## Materials and methods

This single-center, observational study aimed to establish an algorithm using ML to predict the long-term use of iNO (>168 h) and verify its accuracy by classifying patients admitted to the ICU after pediatric open heart surgery at Kagoshima University Hospital.

Ethics approval and consent to participate

The procedures were performed in accordance with the ethical standards of the Committee on Human Experimentation and the 1975 Helsinki Declaration. The study was approved on February 28, 2020, under the title "Investigation of Nitric Oxide Inhalation Therapy in the Perioperative Period of Pediatric Surgery at Kagoshima University Hospital."

This single-center observational study was approved by the Ethics Committee on Epidemiological Studies of Kagoshima University Hospital (approval number: 190250epi). Informed consent was obtained from the patients' guardians.

Patients

An algorithm was established, and its accuracy validated to predict patients with long-term iNO use among 34 pediatric patients who underwent open heart surgery, survived, and were discharged from the ICU of Kagoshima University Hospital between April 2016 and March 2019. Patients’ characteristics are shown in Table 1.

**Table 1:** Explanatory variables in CHD patients. CHD: Congenital heart disease; CPB: Cardiopulmonary bypass time; iNO: inhaled nitric oxide; VIS: Vasoactive-inotropic score; CVP: Central venous pressure; Lac: Lactate value; P/F: PaO_2_/F_I_O_2_; FO: Fluid overload.

Variables	n
Age (months)	7.0 (2.0-16.5)
Height (cm)	71.6 (59.9-81.2)
Weight (kg)	8.37 (5.19-9.99)
BMI (kg/m^2^)	15.2 (14.5-15.7)
BSA (m^2^)	0.4 (0.28-0.46)
RACHS-1 score (n)	
1	2
2	25
3	5
4	2
5	0
6	0
Type of CHD	
Univentricular hearts (n)	16
Biventricular hearts (n)	18
CPB time (min)	168 (123-228)
Aorta cross-clamp time (min)	68 (34.5-122.5)
Total intraoperative blood loss (g)	26.0 (15.0-38.0)
Intraoperative in-out balance (ml)	82.0 (41.0-181.0)
VIS	12.5 (9.25-14.8)
CVP (mmHg) (ICU admission)	14.0 (10.5-16.0)
Lactate (mmol/L) (ICU admission)	1.65 (1.20-3.25)
P/F (ICU admission)	74.0 (50.8-147.8)
Postoperative FO recovery day	2.0 (1.0-3.0)

Patients who used iNO during cardiopulmonary bypass (CPB) and those who died during ICU stay were excluded.

High-flow nasal cannula management

Delivery Systems

Our delivery and monitoring system is mobile. Our facility uses an 800 ppm NO formulation for iNO, using the iNOflo® DS (dosing device) (Mallinckrodt Manufacturing LLC., NJ, USA). For patients on mechanical ventilation, an injector module was connected to the intake line of the ventilator circuit, supplying NO to maintain a constant concentration in the inspiratory air while adjusting the dose proportionally to changes in the ventilator waveform.

An electrochemical analyzer was used to measure the inlet NO and NO2 concentrations using the side stream method proximal to the endotracheal tube. The exhaled gases from the ventilator and the exhaust air from the analyzer were vented away.

Patients undergoing high-flow therapy underwent a procedure similar to tracheal intubation, using a dedicated high-flow therapy device, with NO2 sampled in the same manner for both nasal cannula and mask.

Measurements

All children were monitored with a central venous (CV) catheter inserted during the operation. Pulmonary artery hypertension was defined as an acute rise in pulmonary pressure associated with a decrease in arterial or venous oxygen saturation.

Acute Hemodynamic and Blood Gas Measurements

Heart rate, arterial pressure, central venous pressure (CVP), and arterial blood oxygen saturation were continuously monitored by the care provider. Blood gas analysis was performed every six hours to determine arterial blood oxygen partial pressure, P/F ratio, and lactate level.

Baseline hemodynamic and blood gas measurements were obtained after admission when the patient was stable. NO administration was initiated in all patients.

NO was administered at a concentration of 20 ppm until the patient’s ventilator weaning decision by the care provider, gradually reducing over 6 to 12 hours.

Methemoglobin concentrations were continuously monitored using the iNOflow unit.

ML analysis

We adopted 16 clinical features (Table 1), reflecting patient and surgical characteristics, to predict the patients who needed iNO for over 168 hours using ML analysis. In Japan, medical reimbursement allows the addition of nitric oxide gas for iNO for up to 168 hours during the perioperative period of cardiac surgery, which is calculated on the day this treatment is completed. Therefore, 168 hours was used as the time breakpoint in this analysis.

Adopted Features

The adopted 16 features are shown in Table 1. Risk Adjustment for Congenital Heart Surgery-1 (RACHS-1) classification categorizes individual operative risk, with mortality classified into six categories, indicating that patients with a high RACHS-1 score would have a high index of mortality and morbidities [11]. The vasoactive-inotropic score (VIS) is an upgrade of the inotropic score and quantifies hemodynamic support objectively. VIS was calculated as follows: dobutamine dose (μg/kg/min) + dopamine dose (μg/kg/min) + norepinephrine dose (μg/kg/min) × 100 + epinephrine dose (μg/kg/min) × 100 + milrinone dose (μg/kg/min) × 10 + vasopressin dose (unit/kg/min) × 10,000. Several studies have shown a correlation between high VIS values and poor prognosis [12]. Fluid Overload (FO) indicates the number of days in the postoperative ICU that the patient returned to preoperative weight.

Approach for ML Analysis

Data were stratified according to event and randomly divided into training (80%) and testing (20%) cohorts. To overcome imbalanced data, we applied the synthetic minority oversampling technique for training cohorts [13]. Given the small sample size, it was necessary to reduce the feature set to prevent the influence of overfitting. The decrease in Gini impurity was applied to select important prognostic features [14].

We used AutoGluon, an AutoML framework, to automatically optimize model selection and hyperparameters [15, 16]. This approach allows for efficient exploration of a wide range of models and configurations. There are three steps to proceed with the analysis: (1) stacking: train individual models, (2) bagging, and (3) multilayer stacking: combine the output of each model with the data to perform stacking again, train multiple models on it, and finally use a linear model for output. The specific parameters were bag_folds: 5, stack_levels: 2, and presets: best_quality.

A receiver operating characteristic (ROC) curve was created to analyze the predictive performances of the model, and the area under the ROC curve (AUC) was calculated. Moreover, accuracy, F1 score, precision (positive predictive value), and recall (sensitivity) were also calculated. The F1 score (F score or F measure) is the harmonic average between precision and recall. On the testing data set, the prediction performance of the ML model was evaluated by calculating the above parameters including AUC, accuracy, F1 score, precision, and recall.

## Results

Patient characteristics

As shown in Table 1, the results for patient characteristics include 16 features: age (months), height (cm), weight (kg), BMI (kg/m^2^), BSA (m^2^), RACHS-1 score, type of CHD, CPB time (min), aortic cross-clamp time (min), total intraoperative blood loss (g), intraoperative in-out balance (ml), VIS, CVP, lactate, P/F ratio, and postoperative fluid FO recovery day. Values, other than RACHS-1 score and type of CHD, indicate median (IQR).

ML model for predicting the need for iNO over 168 hours

Among the 28 patients in the training cohort, five needed iNO for over 168 hours; out of six patients in the testing cohort, one needed iNO for over 168 hours.

CPB, aortic clamp time, in-out balance, and lactate were the four most important features for predicting which patients needed iNO for over 168 hours.

Table 2 presents the diagnostic performance of the ML model in the training and testing cohorts for predicting the need for iNO over 168 hours. In the training cohorts, the ML model achieved perfect classification with an AUC of 1.00. In the testing cohort, the ML model also achieved perfect classification with an AUC of 1.00 and an accuracy of 1.00.

**Table 2:** Prediction accuracy. AUC: Area under the receiver operating characteristics curve.

	Accuracy	Precision	Recall	F-measure	AUC
Training cohort	1.00	1.00	1.00	1.00	1.00
Testing cohort	1.00	1.00	1.00	1.00	1.00

## Discussion

The current study evaluated the usefulness of the ML approach using clinical features for predicting the need for iNO over 168 hours in pediatric patients who underwent open heart surgery. The ML model demonstrated great predictive performance with a high AUC. CPB, aortic cross-clamp time, in-out balance, and lactate significantly contributed to the modeling process. Therefore, this ML model using these clinical features can be useful for predicting the need for iNO for a long period in pediatric patients undergoing open heart surgery.

Thirty-four patients were included in this study: 18 with biventricular circulation and 16 with single ventricular circulation. In biventricular circulation, it is important to close the defective hole that may cause a left-to-right shunt and to operate and repair the hemodynamics as early as possible. Conversely, in uni-ventricle circulation, the superior and inferior vena cavae are directly anastomosed to the pulmonary artery. NO is used postoperatively because in biventricular circulation, its vasodilating effect is used to efficiently turn the body circulation and pulmonary circulation, which is expected to reduce pulmonary vascular damage and increase cardiac output.

In uni-ventricle circulation, because there is no pump in the right ventricle, NO is used to efficiently turn the pulmonary circulation by lowering the mean pulmonary artery pressure, allowing arterial blood returning to the single ventricle to be ejected out of the aorta, thereby increasing cardiac output.

Furthermore, in biventricular circulation, there may be damage to the pulmonary vascular bed due to PH associated with a left-to-right shunt, and the elevation of the potent vasoconstrictor endothelin-1 [17] and various inflammatory cytokines [18] suggest that severe inflammation has been induced. Therefore, in biventricular circulation, persistent PH, and long-term and strict circulatory management with iNO may be necessary until reverse remodeling for postoperative PH is achieved.

In cardiac surgery, CPB and aortic cross-clamp time are crucial for achieving a motionless surgical field. However, the use of this intervention in pediatric cardiac surgery is associated with significant inflammation, fluid overload (FO), and end-organ dysfunction resulting in morbidity and mortality [19]. The anti-inflammatory effects [20] and the potential of NO to improve blood congestion and right-sided heart failure suggest that longer CPB and aortic cross-clamp times would likely necessitate prolonged NO therapy.

Regarding intraoperative in-out balance, it has been suggested that CPB-associated vascular endothelial glycocalyx shedding [21] may be facilitated by the release of brain natriuretic peptide, causing vasodilation [22]. Prolonged cardiovascular drug therapy is frequently administered following cardiac surgery with CPB. Severe left ventricular systolic failure, preoperative PH, and postoperative FO have been reported as risk factors [23]. Furthermore, cardiogenic pulmonary edema due to FO following cardiac surgery is recognized as a cause of AKI, subsequent respiratory complications, and pulmonary hypertension, and it is associated with increased mortality rates [24]. Therefore, postoperative iNO is expected to reduce these postoperative complications by suppressing the increase in hydrostatic pressure and the leakage from capillaries into the interstitial space associated with fluid overload, provided that cardiac and renal functions are normal or only mildly impaired.

The use of lactate as an indicator of systemic perfusion in early repair surgery for neonates and infants with CHD has been previously reported [25]. Furthermore, hyperlactatemia has been reported to occur during CPB in patients undergoing surgery for CHD and may be an early indicator of postoperative morbidity and mortality [26].

Therefore, iNO therapy may improve outcomes in patients undergoing cardiac surgery by targeting two major events. First, pre-existing PH and elevated PVR are complex risk factors that affect postoperative morbidity and mortality, primarily through their effects on right ventricular dysfunction and the resulting circulatory failure. Through selective pulmonary vasodilation, iNO can improve pulmonary (and systemic) hemodynamics and potentially prevent low cardiac output syndrome. Second, NO is considered a major regulator of local myocardial ischemia and reperfusion injury, and a modulator of pulmonary and systemic inflammatory responses associated with cardiac surgery. Therefore, iNO may regulate inflammatory outcomes such as myocardial injury and postoperative dysfunction or damage to the myocardium, lungs, or kidneys. In biventricular circulation, the actual mean PVR and PVR values are often higher than those in Fontan circulation. Consequently, vascular damage and inflammation due to PH are more likely to occur, suggesting a longer duration of iNO administration in clinical practice [27]. In the Fontan procedure, the primary hemodynamic characteristic is the lack of a sub-pulmonary ventricle, which automatically leads to a high CVP. This creates additional driving pressure for the pulmonary circulation and diminishes cardiac preload in the systemic ventricle, resulting in chronically low cardiac output, especially in the early postoperative period [28].

Therefore, maintaining a low PVR after pediatric cardiac surgery is essential. Even in cases of intraoperative fluid retention, NO is expected to effectively maintain blood pressure and systemic circulation (with lactate as an indicator of systemic perfusion), except in cases of severe heart failure.

Recent advances in ML approaches have led to an increase in the development of predictive algorithms in the intensive care field [11]. Some have used large population datasets to predict the length of stay, ICU readmission, and mortality rates, as well as the risks of developing medical complications or conditions such as sepsis and ARDS. However, to our knowledge, no study has previously investigated the efficacy of the ML approach for predicting the need for iNO for an extended period in pediatric patients who underwent open heart surgery.

In our study, to prevent the influence of overfitting, we tried to reduce the set of features as much as possible according to the "1:10 rule" [29]. Although we could not reduce the set of features to fewer than four, the ML model with AutoGluon was constructed using the four most important features, including CPB, aortic clamp time, in-out balance, and lactate, which were selected by the decrease in Gini impurity [14]. As previously mentioned, these selected features have been reported to be associated with the demand for iNO in pediatric patients who underwent open heart surgery.

AutoGluon is a framework that attempts to avoid a hyperparametric search to the greatest extent possible by training multiple models concurrently and weighting them using a multilayer stacking strategy to obtain the best predicting model [15, 16]. These processes lead to the optimization of predictive accuracy. In our study, in the training cohort, the ML model also reached perfect classification with an AUC of 1.00. In the testing cohort, the ML model achieved perfect classification with an AUC of 1.00. Therefore, the ML model using these important features can be useful for predicting the need for long-term iNO in pediatric patients who underwent open heart surgery.

Limitations

This study had some limitations. First, it was conducted at a single institution, and the number of cases was small. Second, the ML approach has its limitations. The ML model using AutoGluon was applied solely in the ML analysis. AutoGluon trains a diverse set of models to possibly fuse them, which is an extremely effective strategy [15, 16]. Third, despite achieving good classification performance in training and testing validation, a training-test scheme with a larger population might be preferred for model validation. Fourth, our present study addresses only the survival group, which requires caution in interpretation. Fifth, given that all participants were Japanese, the generalizability of the results to other ethnic groups may be constrained. Finally, the heterogeneous nature of the CHD population could affect the generalization of the findings regarding the impact of predictive factors of iNO administration time within this group.

## Conclusions

An ML approach showed that four factors (CPB, in-out balance, aortic cross-clamp time, and lactate) were strongly associated with long-term iNO administration after open heart surgery in CHD. By understanding the outcomes obtained in this study, we can carefully manage iNO administration in postoperative pediatric patients with CHD and PH, potentially preventing the recurrence of postoperative PH and hypoxemia, thereby contributing to safer management. The ML model using these important features can be useful for predicting the need for long-term iNO in pediatric patients who underwent open heart surgery, but more research is warranted.
